# A Refined Approach to Isolate Interneurons for High-Validity Epigenetic Studies in Human Brain Tissue

**DOI:** 10.3390/mps8030061

**Published:** 2025-06-05

**Authors:** Ariel Cariaga-Martínez, Kilian Jesús Gutierrez, Ignacio Regidor, Marta Del Álamo, Jerónimo Saiz-Ruiz, Raúl Alelú-Paz

**Affiliations:** 1 Medical School, Universidad Alfonso X, 28691 Madrid, Spain; 2 Biological Research Laboratory Professor Giacomo Rizzolatti, Madrid Scientific Park, 28049 Madrid, Spain; 3 Innate Immune Response Group, Hospital La Paz Institute for Health Research, 28046 Madrid, Spain; kilian.jgutierrez@gmail.com; 4 Clinical Neurophysiology Department, Hospital Universitario Ramón y Cajal, 28034 Madrid, Spain; ignacio.regidor@salud.madrid.org; 5 Neurosurgery Department, Hospital Universitario Ramón y Cajal, 28034 Madrid, Spain; marta_delalamo@yahoo.es; 6 Department of Psychiatry, Universidad de Alcala/Hospital Ramón y Cajal, IRyCIS, CIBERSAM, 28034 Madrid, Spain; jeronimo.saiz@salud.madrid.org; 7 Department of Psychology, Universidad Francisco de Vitoria, 28223 Madrid, Spain; 8 School of Experimental Sciences and Technology Biology and Geology Physics and Inorganic Chemistry Department, Rey Juan Carlos University, Tulipán St, s/n, 28933 Móstoles, Madrid, Spain; 9 Hospital Universitario Ramón y Cajal, IRyCIS, 28034 Madrid, Spain

**Keywords:** cortical interneurons, neuronal isolation, human brain tissue, brain epigenetics

## Abstract

Epigenetic research has made notable progress in recent years, yet our ability to explore the human brain at a cellular level remains limited. One of the main obstacles has been the difficulty of isolating specific neuronal populations from postmortem tissue—particularly interneurons, which play a central role in many psychiatric disorders. In this study, we present a practical and reproducible protocol for isolating GAD-positive interneurons from human brain samples. We isolate permeabilized cell-like structures suitable for downstream epigenetic analysis. To ensure specificity, we validated the isolated cells by comparing them with interneurons derived from human iPSCs. This approach allows for high-quality DNA extraction suitable for downstream epigenetic analysis, including methylation-specific PCR. By targeting a well-defined neuronal subtype, our method provides a solid foundation for studying the molecular changes associated with disorders such as schizophrenia and autism. This protocol opens new doors for cell-specific investigations in brain tissue, a step forward in understanding how epigenetic mechanisms contribute to neuropsychiatric pathophysiology.

## 1. Introduction

In recent decades, neuroscience has made significant strides in uncovering the molecular basis of mental health conditions and neurological disorders. Although these conditions are clinically diverse, they often share a common feature: the brain as the biological substrate of the mind and its dysfunctions. Among the many research avenues, epigenetics has gained increasing relevance by highlighting how environmental factors can influence gene expression and potentially contribute to the development of psychiatric disorders [1,2,3,4,5,6]. Understanding these epigenetic mechanisms is particularly important given the global burden of mental health conditions. According to the World Health Organization, depression alone affects more than 300 million people worldwide, with profound social and personal consequences. Gaining insight into how environmental influences interact with genetic predisposition could shed light on the origins of many core symptoms, especially cognitive impairments, which are often the most disabling.

To date, much of the epigenetic research in psychiatry has focused on peripheral tissues, such as blood or saliva [7,8,9,10,11,12]. While these approaches have yielded some valuable findings, they fall short in a crucial aspect: epigenetic regulation is highly tissue-specific and can vary not only between brain regions but also between different cell types within the same region [13,14,15]. This highlights the need for cell-type-specific studies directly in human brain tissue—particularly for key neuronal subtypes like interneurons, projection neurons, and glial cells.

In this regard, interneurons have drawn increasing attention due to their central role in regulating brain circuitry and their involvement in disorders such as schizophrenia, autism, and depression [16,17,18,19,20,21]. These GABAergic neurons are essential for maintaining the balance of excitatory and inhibitory signaling [22,23,24,25,26,27,28,29], and disruptions in their function have been linked to a wide range of neuropsychiatric symptoms. These findings have highlighted the need to develop specific epigenetic studies on interneurons that will allow us to obtain essential information on the molecular mechanisms that cause these conditions.

However, it is not a simple task; despite their importance, isolating specific neuronal populations from human brain tissue remains technically challenging. Existing methods are often resource-intensive or lack the precision required for cell-type-specific epigenetic analysis [30,31,32,33,34,35,36,37,38]. Previous efforts to isolate GABAergic neurons from human brain tissue have predominantly relied on fluorescence-activated nuclear sorting (FANS) techniques, using nuclear markers to distinguish GABAergic from glutamatergic populations [38]. While powerful, these protocols typically require large tissue quantities, ultracentrifugation steps, and specialized multi-omic platforms, making them less accessible for laboratories with limited resources. In contrast, our approach is based on the isolation of permeabilized cell-like structures rather than isolated nuclei, enabling the simultaneous analysis of nuclear and cytoplasmic proteins and facilitating morphological validation. This method reduces the tissue requirement to as little as 0.1 g and eliminates the need for ultracentrifugation, thereby streamlining the workflow and broadening its applicability in resource-limited settings.

In this context, the development of a reliable, accessible protocol for interneuron isolation represents a key step forward. The present study addresses this gap by introducing and validating a new method to obtain high-purity interneuron populations from postmortem brain tissue, paving the way for more accurate and meaningful investigations into the epigenetic underpinnings of mental illness.

## 2. Materials and Methods

### 2.1. Samples

Eight frozen postmortem brain samples were obtained from the dorsolateral prefrontal cortex (DPC) of healthy male subjects, aged between 46 and 81 years, divided into two age groups (<50 and >50 years) (see Table 1). To minimize biases and potential confounding factors, only samples with a postmortem interval of up to 16 h were selected. Brain regions were consistently sampled in each individual using the *Atlas of the Human Brain* [39]. All samples were acquired with written informed consent from the subjects and were included in the study following approval by the ethical committee of the Brain Bank at the University Hospital Alcorcón Foundation and Ramón y Cajal University Hospital (code 335/14), Madrid, Spain. At least 1 cm^2^ or 0.1 g of brain tissue from each sample was processed through the entire protocol, from enzymatic treatment to the final step of cell sorting. While our protocol differs from conventional nuclear isolation methods by enabling the separation of permeabilized cellular structures that retain both nuclear and cytoplasmic components, it is important to clarify that we cannot guarantee the preservation of complete neuronal integrity. Given the steps required for sample preparation, it is likely that subcellular interactions and membranous continuity are partially disrupted. Thus, although the structures obtained go beyond isolated nuclei, they should not be strictly interpreted as fully intact cells.

To ensure demographic diversity and avoid potential systematic age bias, postmortem brain samples were grouped into two broad age categories: individuals younger than 50 years and those older than 50 years. This stratification was applied for descriptive purposes only; no comparative analyses between age groups were conducted as part of the study.

We present below the phases established in the method we developed. We begin with the collection of fibroblasts through a skin biopsy, followed by reprogramming these cells into induced pluripotent stem cells (iPSCs). The iPSCs are then cultured and differentiated into interneurons using a specified protocol. Brain tissue samples undergo enzymatic treatment and gradient centrifugation to isolate distinct cell populations. The isolated cells are then processed for flow cytometry and sorting, with DNA subsequently extracted, modified, and amplified for methylation-specific PCR analysis. Antibody staining and confocal microscopy are used to confirm cell identity and differentiation. Finally, statistical analyses are conducted to validate the findings.

### 2.2. Cell Culture and Differentiation

Fibroblasts were obtained through a skin punch biopsy from the forearm. Induced pluripotent stem cells (iPSCs) were then derived from these fibroblasts by the Spanish National Cell Lines Bank Facility (Stem Cells Unit, Barcelona, Spain) using a non-integrative reprogramming method. The same facility conducted the characterization and verification of stemness to ensure the quality and pluripotency of the iPSCs.

iPS cells were cultured in E8 medium (ThermoFisher, Cat. A1516901, Waltham, MA, USA) using 0.33 mg/mL Matrigel© Reduced-Growth Factor (Corning, Cat. 354230, Corning, NY, USA) as a supportive surface, prepared according to manufacturer’s instructions. Specific differentiation to interneurons was carried out by applying the protocol described by Liu et al. (2013) [40]. Briefly, iPS cells were cultured in suspension for 6 days to generate embryoid bodies. Then, embryoid bodies were cultured onto 60 mm culture dishes and were allowed to adhere in the presence of NIM medium for 6 days. One day later, colonies not forming neural rosettes were eliminated under microscope observation and the remaining formed neural rosettes were treated with 1.5 μM of purmorphamine (Sigma, Cat. SML0868, St. Louis, MO, USA; hereinafter, PUR) for 6 more days. Then, they were detached and suspended to generate neurospheres in the presence of 1 μm of PUR for 6 more days. After that, neurospheres were seeded onto a collagen matrix (0.45 μg/cm^2^) (ThermoFisher, Cat. 23017-015) in the presence of NDM medium supplemented with BDNF (10 ng/mL; Sigma, Cat. B3795), GDNF (10 ng/mL; ThermoFisher, Cat. PHC 7041), IGF (0.1 μg/mL; ThermoFisher, Cat. PMG 0075) and cAMP (1 μm; Sigma, Cat. D0260). This was considered “Day 0” for counting differentiation. Where indicated, at least 60 days of differentiation were required to proceed with the experimentation.

### 2.3. Cell Separation

One milliliter of Accutase™ (Sigma, Cat. A6964, St. Louis, MO, USA) was added to 1 cm^2^ or 0.1 g of brain tissue and incubated for 15 min at 4 °C (a blade was used to cut and to facilitate the enzymatic action). After incubation, the homogenate tissue was strained through a 100-micron-pore-diameter cell strainer (Corning, Cat. 352360, Corning, NY, USA). Filtrate was collected and applied through a 40-micron-pore-diameter cell strainer (Falcon, Cat. 352340). The resulting homogenate was centrifuged for 2 min at 300× *g* (at room temperature to diminish Accutase™ activity) in a swinging-bucket rotor centrifuge (Hamburg, Germany, Eppendorf, Cat. 5702). To collect all the remaining structures, the supernatant was centrifuged again under the same conditions. Finally, a centrifugation step was added to fully recover all the cellular structures (2 min at 1000× *g*). The three pellets were pooled and applied to a column of 3 mL of 20% Percoll^®^ and centrifuged for 3 min at 1000× *g*. The resulting pellet (0.3 mL aliquots) was applied to a column of 0.8 mL of 35% Percoll^®^ and centrifuged for 30 min at 400× *g*. The pellets were washed with 1 mL of PBS 1X and subject to downstream applications. Full details of the procedure are provided in the Supplementary Materials.

### 2.4. Antibodies

All the antibodies were commercially acquired and prepared in the appropriate blocking solution. In the order indicated in the Results section, the following antibodies were used:

- Anti-NeuN Alexa Fluor™ 488 conjugated (Millipore, Cat. MAB377X, Burlington, MA, USA); 1/50 for all experiments.
- Anti-NeuN (clone 60) (Millipore, Cat. MAB377, Burlington, MA, USA); 1/350 for all experiments.
- Anti-GAD 65/67 (Sigma, Cat. G5163, St. Louis, MO, USA); 1/1000 for experiments involving tissues and 1/250 for experiments involving cells.
- Anti-FOXG1 (Abcam, Cat. Ab18259, Cambridge, UK); 1/500 for all experiments.
- Antio-TTF1 (Abcam, Cat. Ab76013, Cambridge, UK); 1/200 for all experiments.
- Anti-Class III β Tubulin (MO, USA, Sigma, Cat. T8660); 1/500 for all experiments.
- Goat anti-rabbit (Alexa Fluor™ 488, ThermoFisher, Cat. A11034, Waltham, MA, USA); 1/500 for confocal microscopy experiments and 1/1000 for flow cytometry assays.
- Goat anti-mouse (Alexa Fluor™ 647, ThermoFisher, Cat. A21236, Waltham, MA, USA); 1/500 for confocal microscopy experiments and 1/1000 for flow cytometry assays.

### 2.5. Cytometry Analysis and Cell Sorting

For flow cytometry assays and/or subsequent cell sorting, after cells were obtained from tissue samples or cultures, they were fixed with 4% paraformaldehyde (Aname, Cat. 15710, Madrid, Spain). Ampules of pre-made 16% methanol-free solution at pH 7.4 were tested for 10 min, and permeabilization was carried out by using 0.1% TritonX100 (Panreac, Cat. A4975, Barcelona, Spain) in PBS 1X for 10 min. Normal goat serum (10% in PBS 1X. Millipore, Cat. S26, Burlington, MA, USA) was used as a blocking reagent (30 min). Appropriate primary antibodies were then added and incubated at 4 °C in a rotating shaker. Fifteen microliters of Fc Receptor Binding Inhibitor antibody (ThermoFisher, Cat. 14-9161-71, Waltham, MA, USA) were added and incubated for 20 min. The cells were washed with 1 mL of PBS1X and appropriated secondary antibodies were incubated for 5 min protected from light. The cells were then washed twice using 1 mL of PBS 1X and subjected to flow cytometry/sorting assays. Unless otherwise indicated, the steps were carried out in a rotating shaker and at room temperature. 

Flow cytometry data was acquired by using a BD FACSCanto II flow cytometer (Beckton Dickinson, Franklin Lakes, NJ, USA). BD FACSAria Fusion (BD Biosciences, Franklin Lakes, NJ, USA) cell sorter was used to separate stained cells. All data was obtained under standard procedures at the Flow Cytometry Unit (Severo Ochoa Molecular Biology Center, Madrid, Spain) and analyzed with BD FACSDiva (v9.0) and FlowJo (v11), LLC softwares.

### 2.6. DNA Extraction, Modification and Amplification

DNA was extracted using the PicoPure^®^ DNA Extraction Kit (ThermoFisher Scientific, Cat. KIT0103, Waltham, MA, USA). Briefly, cell fractions from sorting were collected in 20 microliters of lysis buffer. Two microliters of Proteinase K was added, and digestion was carried out for 18 h at 60 °C. Digested fractions were assayed for DNA concentration by using Qubit 3 technology (ThermoFisher Scientific, Cat. Q32851, Waltham, MA, USA). The average DNA concentration was 0.425 ng/μL (sample 1: 1.21 ng/μL; sample 2: 0.053 ng/μL; sample 3: 0.322 ng/μL; sample 4: 0.038 ng/μL; sample 5: 0.537 ng/μL; sample 6: 0.359 ng/μL). Qubit technology is very accurate for assessing very low concentrations of DNA but does not allow its quality to be assessed; therefore, A260/A280 absorbance ratios were obtained by using NanoDrop 2000 spectrophotometer (ThermoFisher, Waltham, MA, USA), with average ratios of 1.8. An indirect quality measure was also obtained by gel electrophoresis.

DNA was modified by using the Zymo Research Methylation Gold Kit (Zymo Research, Cat. D5006, Irvine, CA, USA) according to the manufacturer’s instructions. Modified DNA (average: 2.5 ng) was used as a matrix for a methylation-specific PCR to amplify the Glutamate Metabotropic Receptor 3 (GRM3 gene, Entrez Gene: 2913) promoter. Specific primers were obtained by using the Methyl Primer Express™ software (v1.0). Selected primers are indicated in Table 2.

PCR was carried out using the Bioline Kit (Bioline, Cat. BIO-21047, Telstar Nurseries, UK, in buffer solution, 1 mMMgCl2, 10 pmoles of each specific primer, 0.4 mM of dNTPs (Bioline, Cat. BIO-39053, Telstar Nurseries, UK) and 1 unit of Taq polymerase in 25 μL reactions). Amplicons were resolved by using a 2% agarose gel electrophoresis. Images were taken by using a GelDoc EZ Imager System (BioRad, Hercules, CA, USA).

### 2.7. Primer Validation and Controls

To validate the potential amplifications observed in genomic DNA obtained through the protocol described here, two commercial DNA samples were utilized: one fully methylated across all cytosines and others completely unmethylated (Zymo Research, Cat.: D5014, Irvine, CA, USA). These commercial DNA samples included manufacturer-supplied primers specifically designed to detect their respective methylation states. This setup allowed us to evaluate the performance and specificity of the custom primers we developed in-house using the Methyl Primer Express™ software (v1.0). The validation strategy, including detailed conditions and outcomes, is outlined in Table 3.

Of the eight postmortem brain samples processed through the full protocol, only six were used for methylation-specific PCR (MSP). We deliberately excluded the samples that yielded the highest and lowest DNA concentrations, as these were considered outliers. This decision was made to ensure that the methylation analysis was based on samples with more representative and consistent performance.

### 2.8. Confocal Microscopy

To obtain confocal microscopy images, sterile glass coverslips were put into dishes and coated with laminin, and neurospheres were then seeded onto them. The differentiation process was follow as indicated. Cells were fixed with 4% paraformaldehyde (pH 7.4) for 10 min and permeabilization was carried out using 0.3% Triton in PBS 1X for 5 min. Normal goat serum (10% vol/vol in PBS 1X) was used as a blocking reagent (30 min). Appropriated primary antibodies were added and incubated overnight at 4 °C, and appropriated secondary antibodies were incubated for 30 min in a dark chamber. Unless otherwise indicated, the steps were carried out at room temperature. Coverslips were mounted (ProLong Mounting Media with DAPI, ThermoFisher, Cat. P36941, Waltham, MA, USA) and images were taken by using a Nikon A1R+ confocal microscope (40X/1.3 oil and 60X/1.4 oil objectives) under standards procedures at the Optical and Confocal Microscopy Facility (Severo Ochoa Molecular Biology Center, Madrid, Spain). The image analysis was carried out with FIJI/ImageJ v2 (https://imagej.net/software/fiji/, accessed on 1 June 2025).

### 2.9. Statistical Analysis

All statistical analyses were carried out by using R software (v4.5) and the chosen significance level was α = 0.05 for applied statistical test (*t*-test or Mann–Whitney test when parametric assumptions were not met). The criteria for significance (α) were set at * *p* < 0.05, ** *p* < 0.01, *** *p* < 0.001, **** *p* < 0.0001.

## 3. Results

### 3.1. Enzymatic Digestion and Differential Density Gradients Allow Adequate Separation of Brain Tissue Cells

**Figure 1:**
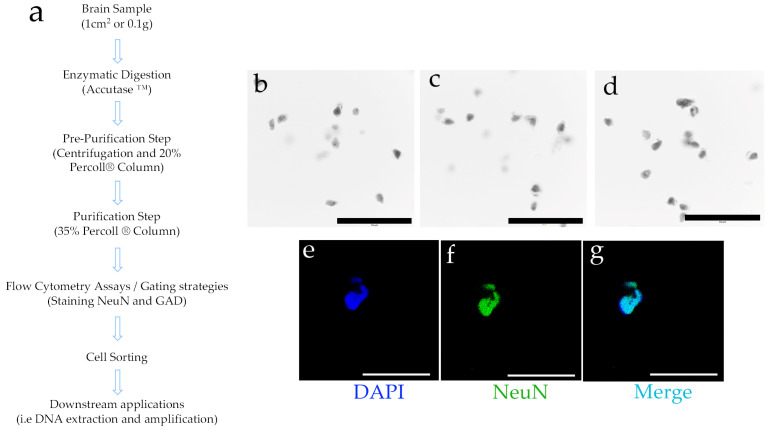
(**a**) Brief description of the main steps of the protocol for cell separation from brain tissue. See Section 2 for a detailed description (further details can also be found in the Supplementary Materials). (**b**–**d**) Three representative light microscopy fields of the obtained results. The obtained cell structures were stained with Trypan Blue and observed (10X); (**e**–**g**) Subsequently, and using DAPI as nuclear dye (**e**), the structures were stained to observe the neuronal marker NeuN (**f**), and a combination of signals was generated (merge, (**g**)). Black or white bars at the bottom = 50 microns.

Starting with human brain tissue samples up to 1 cm^2^ from the DPC, we employed enzymatic digestion followed by centrifugation with fixed concentrations of Percoll^®^ (Figure 1a). This process successfully separated structures resembling neurons and neuronal nuclei. Using Trypan Blue staining—applied solely for microscopy contrast rather than as a cell membrane permeability test, given that the tissue cells were non-viable—distinct structures resembling individual nuclei were visualized. These nuclei displayed prominent nucleoli and characteristic neuronal extensions. Additionally, some observed structures had a morphology reminiscent of pyramidal neurons, a shape typical of certain neuronal types (Figure 1b–d).

Several neuronal populations express the neuronal nuclear protein NeuN, a transcription factor [41]. Therefore, the separated cell structures were subjected to NeuN staining (Figure 1f), showing colocalization with the nuclear staining achieved by DAPI (4′,6-diamidino-2-phenylindole) (Figure 1g). These findings indicate that the structures isolated through this protocol are reactive to a nuclear marker specific to neurons. To further refine our characterization, we aimed to better define the obtained cell subtypes by employing flow cytometry and subsequent cell sorting techniques.

### 3.2. Cell Separation by Using Differential Densities May Help to Purify and Concentrate Cell Populations of Interest

**Figure 2:**
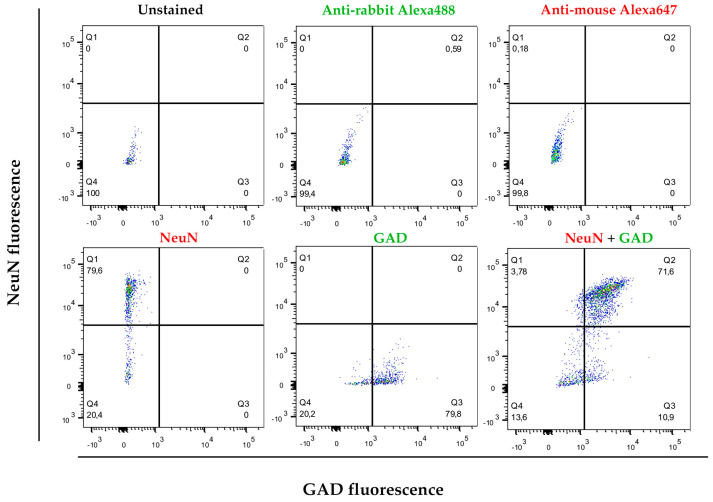
Representative flow cytometry experiment and gating strategy after cell separation using brain tissue. After separation and fixation of the separated cells using the described protocol, they were stained with DAPI as nuclear dye. An anti-NeuN antibody and an anti-GAD antibody were used. The *Y*-axis represents the level of fluorescence for NeuN (Alexa Fluor™ 647) and the *X*-axis represents the fluorescence for GAD (Alexa Fluor™ 488). The population that was positive for DAPI (nuclei) and also positive for NeuN (neurons) and GAD were considered compatible with interneuron markers.

As highlighted in the Introduction, the molecular biology of interneurons is essential for understanding the functionality of numerous brain circuits impacted by various pathologies. Interneurons form local circuits with short projections and, despite comprising different subpopulations with specialized roles, they all function as inhibitory neurons, releasing the neurotransmitter GABA. This neurotransmitter is synthesized through the enzymatic activity of glutamic acid decarboxylase (GAD). To characterize neuronal structures, the nuclear marker NeuN was employed, while interneurons were identified as the cell population positive for both NeuN and GAD. Of all nuclear structures isolated through this protocol (DAPI-positive), an average of 64.08% (CI = 53.93–74.23%; α = 0.05; *n* = 6) were NeuN-positive. Upon double staining, an average of 56.16% (CI = 44.98–67.35%; α = 0.05; *n* = 6) of nuclei were positive for both NeuN and GAD (NeuN+/GAD+), identifying them as interneurons (Figure 2).

These results underscore the importance of using differential density gradients to effectively concentrate cell populations based on distinct morphological characteristics. This method not only allows for precise cell separation but also enhances the quality of isolated cells for further molecular analyses. Importantly, DNA extracted from these selectively enriched samples is of sufficient quality for downstream applications, including PCR amplification and methylation-specific PCR, thereby expanding the potential for in-depth epigenetic and molecular studies.

### 3.3. The Obtained Genetic Material Can Be Used for Downstream Applications, in This Case, Methylation-Specific PCR

**Figure 3:**
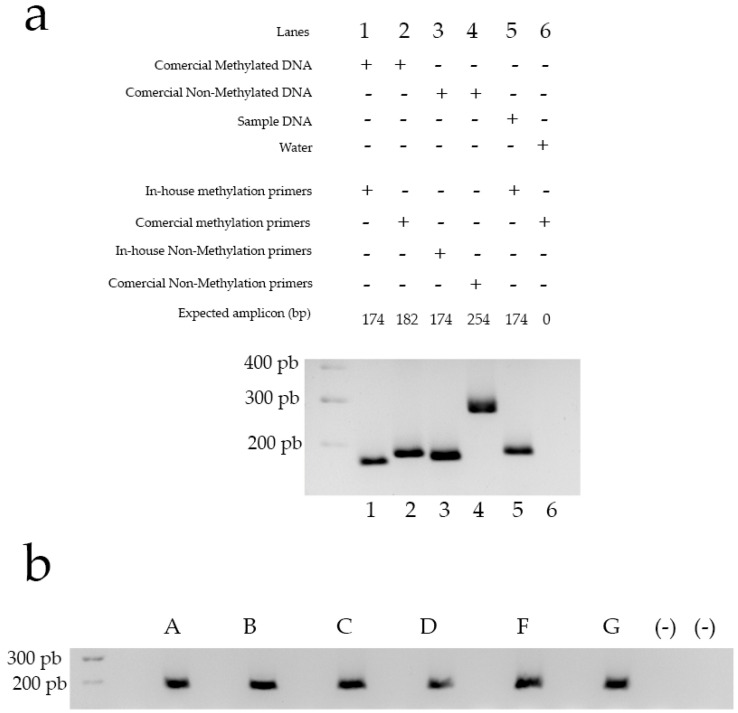
(**a**) Control strategy for MSP. Lanes 1–6 show obtained amplicons according to Table 3 strategy (see Material and Methods). (**b**) Methylation-Specific PCR. Lanes show the amplification product of a methylation-specific PCR (primer pairs to determine methylation) using DNA obtained after cell sorting of six samples (A to F) from prefrontal cortex (brain tissue). The expected molecular weight of the amplification product is 174 bp. (-): negative controls where water was used instead of DNA samples.

Following separation and cell sorting, the DNA obtained is suitable for various downstream applications. To demonstrate this capability, we isolated DNA from six samples after applying the fractionation protocol developed in this study, followed by cell sorting. This DNA was then used as a template for methylation-specific PCR, employing custom-designed primers to assess the methylation status of the GRM-3 gene promoter (Figure 3). As controls for primer specificity, fully methylated and unmethylated commercial DNA samples were used. The results, based on the control strategy, illustrated in Figure 3, panel A, confirm that the primers are sufficiently specific to reliably detect methylation states in DNA derived from brain tissue processed with our protocol. These findings validate the protocol’s efficacy for precise epigenetic analysis in neuronal samples, highlighting its potential for use in targeted studies of DNA methylation in specific neuronal populations. The successful detection of methylation states in isolated cell types demonstrates the protocol’s utility for advancing our understanding of cell-type-specific epigenetic mechanisms, which may be crucial in uncovering the molecular underpinnings of neurological and psychiatric disorders.

### 3.4. The Criteria Developed in This Protocol Can Be Contrasted by Using Interneurons Differentiated from Adult Induced-Pluripotent Stem Cells (iPSC)

One of the primary technical challenges in staining cells from tissue samples, particularly with fluorescent markers, is tissue autofluorescence. This effect is especially pronounced in fatty tissues like the brain, where it can interfere with specific labeling and obscure results. To validate the unusually high concentration of structures reactive to interneuron markers observed after applying our protocol, we opted to differentiate interneurons from iPSCs, focusing specifically on NeuN and GAD markers to determine if results obtained with our cell fractionation method could be replicated in a control model of differentiated interneurons.

**Figure 4:**
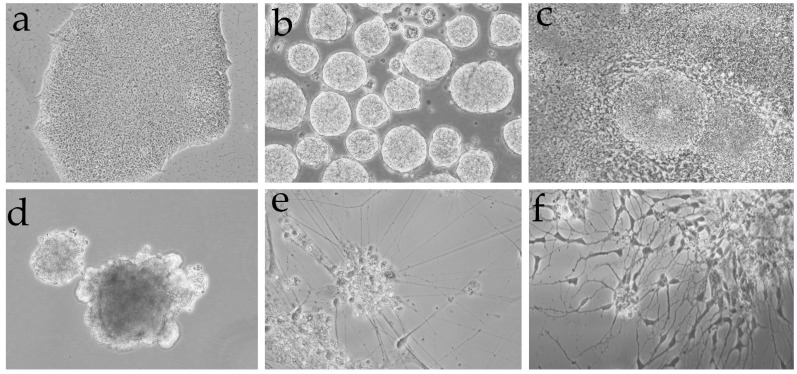
Brief summary of the differentiation procedure from iPSCs to neurons. (**a**) iPSC cell colony; (**b**) embryoid bodies formed by developing iPSC clumps in suspension; (**c**) neural rosettes which is an early stage and where PUR treatment is initiated; (**d**) after several days at high concentrations of PUR, neural rosettes are detached and progenitors (neurospheres) develop in suspension culture; (**e**,**f**) in the presence of PUR and after seeding onto a collagen matrix and in the presence of growth factors, neurospheres generate the structural nuclei of mature neuronal colonies.

Following the differentiation protocol described by Liu et al. [40], we generated interneurons from iPSCs (Figure 4). Initially, iPSCs grow as distinct colonies with well-defined borders and display a high nucleus-to-cytoplasm ratio (Figure 4a). These iPSCs are then cultured in suspension to form embryoid bodies (Figure 4b), which are subsequently seeded to develop neural rosettes (Figure 4c). After treatment with a high concentration of purmorphamine (PUR), which robustly activates the Sonic Hedgehog signaling pathway for patterning, the neural rosettes are collected under microscopic guidance and cultured in suspension to form neurospheres (Figure 4d). These neurospheres are then seeded onto a collagen matrix and allowed to differentiate for a minimum of 60 days in the presence of PUR to complete the process. This model allows us to accurately assess the specificity of our protocol in generating and identifying interneuron populations through precise marker labeling.

**Figure 5:**
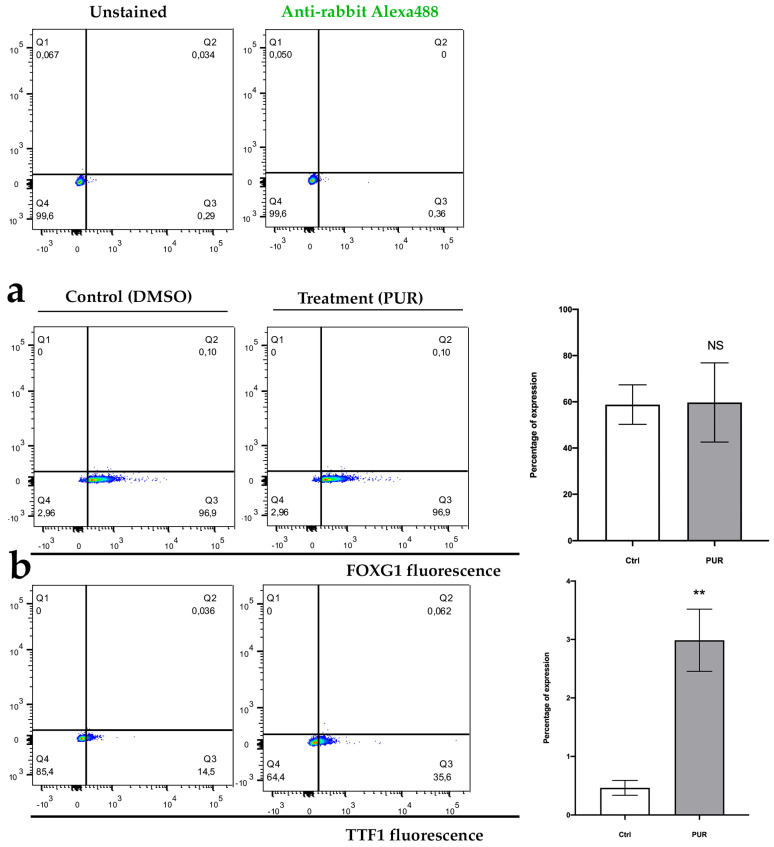
(**a**) Representative experiment of gating strategy for FOXG1 staining and (**b**) TTF1 staining, in cells obtained after disaggregating neurospheres. NS = not significant; ** *p* < 0.05; *n* = 3.

As a first step in characterization using neurospheres (progenitors), the expression of FOXG1 (a transcription factor and widely used as a marker of cortical neuronal lineage [42,43] and TTF1 (also a transcription factor, commonly used as a marker of future interneuron lineage [44,45] was assessed. The results indicate that after a mean of 8.3 (±4.5) days of PUR treatment, 58.8% (CI = 49.11–68.48%; α = 0.05; *n* = 3) of cells from the control neurospheres and 59.7% (CI = 40.31–79.08%; α = 0.05; *n* = 3) of cells from PUR-treated neurospheres were positive for the FOXG1 marker. This difference was not statistically significant (*p* > 0.05) (Figure 5a). Contrarily, after a mean of 6 (±3) days of PUR treatment, 0.46% (CI = 0.31–0.60%; α = 0.05; *n* = 3) of cells from control neurospheres and 3% (CI = 2.43–3.57%; α = 0.05; *n* = 3) of cells from PUR-treated neurospheres were positive for the TTF1 marker. This difference was statistically significant (*p* < 0.05), showing a large effect size (Cohen’s d > 0.8) (Figure 5b). These results indicate that PUR treatment is effective to generate progenitors whose lineage will eventually generate interneurons.

**Figure 6:**
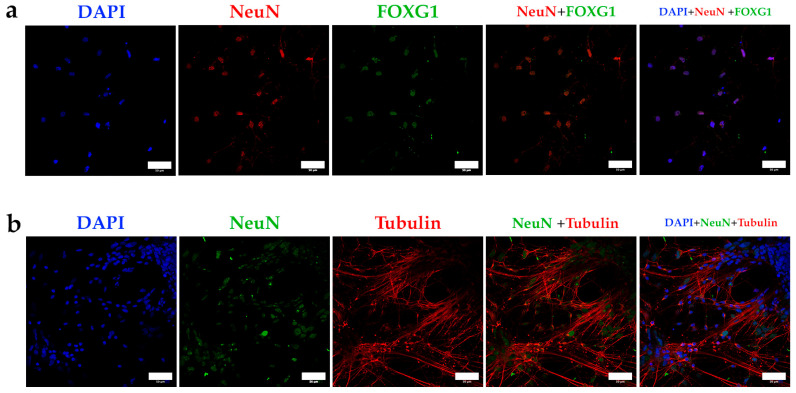
(**a**) Confocal microscopy images obtained by staining the cortical marker FOXG1 in differentiated neurons, obtained after applying the differentiation protocol for at least 60 days; (**b**) confocal microscopy images obtained by staining class III β-tubulin, an isoform presents almost exclusively in neurons. Bars = 50 microns.

To confirm the cortical and interneuronal identity of the differentiated neurons, we used a panel of specific markers: FOXG1 as a cortical marker, NeuN (nuclear) and Class III β-tubulin (cytoplasmic, referred to as tubulin) as neuronal markers [40,46], and GAD as a marker for interneurons. A summary of the staining results is presented in Figure 5. The differentiation protocol successfully produced cells reactive to NeuN, which colocalizes with the cortical transcription factor FOXG1 in the nuclei (Figure 6a). Additionally, the expression of Class III β-tubulin confirms neuronal identity (Figure 6b).

**Figure 7:**
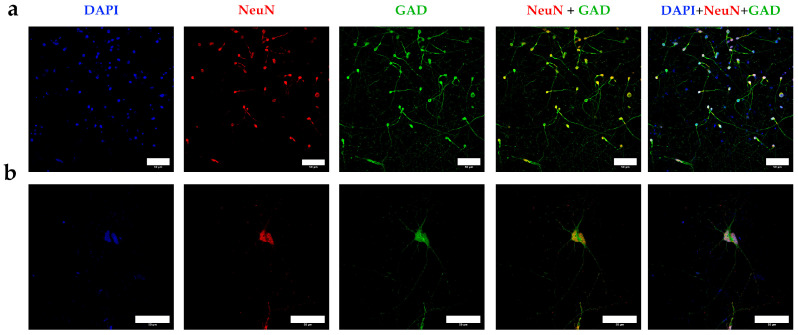
(**a**) Confocal microscopy images obtained by staining the neuronal marker NeuN and using GAD as an interneuron marker, in differentiated neurons obtained after applying the differentiation protocol for at least 60 days (60X); (**b**) A higher magnification of the previous images to highlight the structure and markers of putative interneurons obtained in culture (60X). Bars = 50 microns.

**Figure 8:**
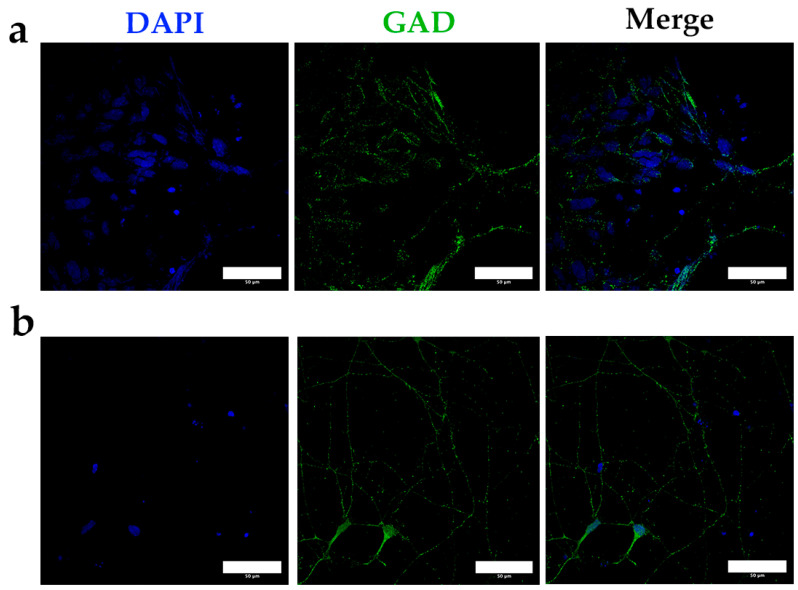
(**a**) Confocal microscopy images obtained after specific staining of GAD as an interneuron marker, in differentiated neurons obtained after applying the differentiation protocol for at least 60 days in control neurons (40X). (**b**) Confocal microscopy images obtained after specific staining of GAD as an interneuron marker, in differentiated neurons obtained after applying the differentiation protocol for at least 60 days in PUR-treated neurons (40X). Bars = 50 microns.

After a minimum of 60 days of differentiation, the neurons displayed characteristic morphology, with compact nuclei, a rounded cell structure, and numerous short cytoplasmic extensions (Figure 7a). At higher magnification, thinner and more branched extensions were observed, along with nuclear GAD localized in discrete punctate patterns (Figure 7b). This specific staining pattern was absent in the control samples (Figure 8), further validating the effectiveness of our differentiation protocol in generating neurons with a clear cortical and interneuronal identity.

These results indicate that treatment with PUR generates progenitors whose lineage is cortical and, after differentiation, express markers of interneuronal lineage. The differentiated neurons maintain this cortical character, while expressing nuclear and cytoplasmic markers characteristic of neurons. In addition, mature neurons show morphology and nuclear and cytoplasmic markers compatible with those described for interneurons.

### 3.5. The Markers Applied to the Separation of Populations in Brain Tissue Samples Separate a Discrete Population in Cultures of Interneurons Obtained by Reprogramming

**Figure 9:**
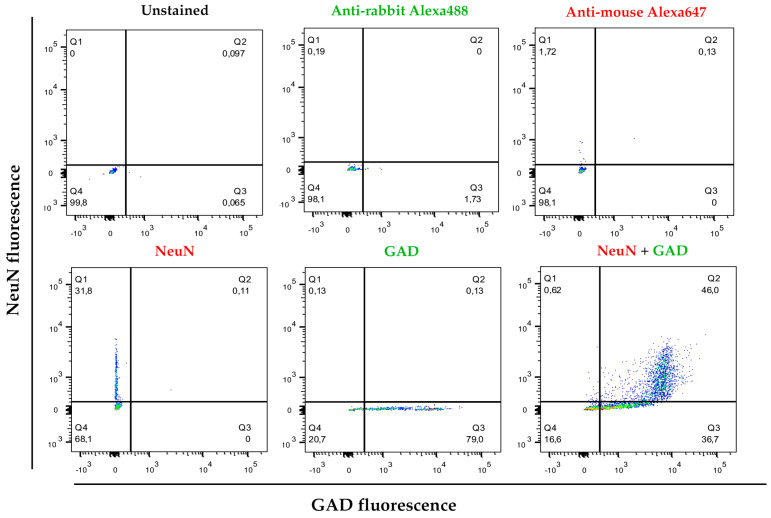
Representative flow cytometry experiment and gating strategy for differentiated neurons obtained after applying the differentiation protocol for at least 60 days. After cell fixation, they were stained with DAPI as nuclear dye. An anti-NeuN antibody and an anti-GAD antibody were used. The *Y*-axis represents the level of fluorescence for NeuN (Alexa Fluor™ 647) and the *X*-axis represents the fluorescence for GAD (Alexa Fluor™ 488). The population that was positive for DAPI (nuclei) and also positive for NeuN (neurons) and GAD were considered compatible with interneuron markers.

As a final validation step, the markers used for cell separation in brain tissue were applied to a pure culture of neurons after their differentiation into interneurons. In cultures with 75 ± 4 days of differentiation, an average of 65.5% (CI = 56.85–68.14%; α = 0.05; *n* = 5) of DAPI-positive events also expressed both NeuN and GAD markers (NeuN+/GAD+ population) (Figure 9). These findings demonstrate that the markers selected for isolating cell populations using our protocol can effectively identify a distinct population within cultures enriched with differentiated neurons, specifically those expressing characteristic markers of interneuron populations. This consistency further supports the robustness of our protocol in distinguishing relevant neuronal subtypes in both brain tissue and controlled culture conditions.

## 4. Discussion

This study presents a practical and efficient protocol for isolating and enriching interneuron-like populations from human brain tissue. By combining density-based separation techniques with a validated set of molecular markers, we were able to identify a subset of neurons consistent with the phenotype of GABAergic interneurons. Importantly, we confirmed the specificity of our method by applying the same markers to cultured neurons derived from iPSCs, demonstrating a high degree of overlap between the two systems. This cross-validation underscores the reliability of our protocol in distinguishing interneurons in both native and in vitro settings.

Our method offers a simplified and accessible approach for isolating GABAergic interneurons from small postmortem human brain samples, in contrast to the more technically demanding nuclear sorting protocols used by other groups. For instance, Kozlenkov et al. [38] and Kozlenkov et al. [47] employed fluorescence-activated nuclei sorting (FANS) with SOX6 and NeuN to distinguish GABAergic from glutamatergic neurons and oligodendrocytes, followed by multi-omic profiling. These protocols, while powerful, require large tissue amounts, ultracentrifugation, and advanced analytical platforms.

In contrast, our protocol isolates fixed, permeabilized whole cells, enabling simultaneous staining of nuclear and cytoplasmic proteins and visualization of cell morphology. The method requires only ~0.1 g of tissue, uses readily available reagents, and can be implemented in standard molecular laboratories. Additionally, we validated our marker strategy using iPSC-derived interneurons, further supporting its applicability across models. While we acknowledge that our approach does not resolve interneuron subtypes at the molecular level, it provides a robust and reproducible method to isolate GAD-positive neurons suitable for downstream DNA-based analysis in resource-limited settings, for example, in DNA methylation studies. We selected GRM3 as a proof-of-concept target due to its known epigenetic relevance in mental illness [48,49,50,51,52]. Although its expression may be relatively low in some neuronal subtypes, our objective was to confirm that the DNA isolated through our protocol is suitable for methylation-specific amplification. DNA yield per sample averaged 0.425 ng/µL, sufficient for multiple downstream PCR reactions, and the methylation-specific primers were validated using commercially methylated and unmethylated controls.

Access to human brain tissue is essential for advancing our understanding of psychiatric disorders, especially those with strong environmental components such as schizophrenia and bipolar disorder [53]. However, the brain’s cellular complexity poses a major obstacle—different neuronal subtypes, such as projection neurons and interneurons, exhibit distinct epigenetic signatures [38]. In brain regions relevant to psychiatric research, like the prefrontal cortex, interneurons represent only a small fraction of the neuronal population, making their isolation particularly challenging [54]. This problem has been solved by increasing sample sizes, which forces us to obtain a large amount of tissue [55,56,57], which may be feasible in animal models but are rarely practical when working with valuable human samples. Compared to earlier methods, such as those described by other authors, our protocol minimizes sample requirements and simplifies processing without sacrificing specificity. Although structural preservation is limited, this trade-off is common across many molecular protocols—including advanced proteomics techniques—when working with small simples [58]. Additionally, by validating the use of NeuN and GAD markers across both postmortem brain samples and iPSC-derived interneurons, we offer a robust strategy for cross-model comparison. It is important to note that the primary goal of our study was not to subclassify GABAergic interneurons but to develop a protocol that isolates a high-purity NeuN+/GAD+ population compatible with DNA-based applications. While single-cell RNA-seq could offer deeper insights into interneuron diversity, it is often not feasible when working with limited postmortem tissue. Future studies may expand on our work by applying transcriptomic or epigenomic profiling to further characterize these populations.

In this regard, it is worth noting that the proportion of NeuN+/GAD+ cells isolated from postmortem samples (56%) is higher than the expected physiological proportion of GABAergic neurons in the dorsolateral prefrontal cortex, which is typically estimated at 20–30%. This discrepancy is likely the result of the enrichment effect generated by our protocol, which combines sequential density gradients and antibody-based gating. This methodological strategy selectively enhances the recovery of interneuron-like cells relative to the original tissue composition.

But this is not the only difficulty we have faced when working with human samples: autofluorescence is another common hurdle when labeling brain tissue, due to the high lipid content of the human brain. Although various approaches have been proposed to reduce autofluorescence [59,60], they often require large amounts of tissue or extended processing times. By using density gradients and antibody-based flow cytometry, we were able to circumvent these issues and successfully isolate cell populations with high specificity, even from small samples.

To further validate the protocol, we turned to a controlled iPSC-derived model of interneuron development. Using a well-established differentiation protocol that mimics the generation of cortical interneurons via Sonic Hedgehog pathway activation [40], we obtained cultures enriched in cells expressing markers such as FOXG1, TTF1, NeuN, and GAD. These results confirmed that purmorphamine treatment reliably drives progenitors toward an interneuronal lineage, and that the combination of markers used in our protocol can successfully identify these cells in culture as well.

The consistency between our results from postmortem brain tissue and the iPSC-derived model lends strong support to the specificity and robustness of our method. Nevertheless, future work could include additional controls—such as the inclusion of projection neurons or glial markers—and more comprehensive epigenetic profiling, for instance through epigenome-wide association studies (EWAS), to further validate the identity and purity of the isolated populations.

Studying the molecular basis of psychiatric disorders directly in human brain tissue remains a major challenge, particularly given the scarcity of samples and the heterogeneity of the brain. Bulk tissue analyses often obscure critical cell-type-specific differences, leading to misleading conclusions. Our protocol offers a practical solution: it enables rapid, cost-effective, and reproducible isolation of specific neuronal populations from limited human tissue, making it well-suited for high-validity epigenetic studies. By narrowing the focus to defined cell types such as interneurons, this approach holds significant potential for clarifying the cellular and molecular mechanisms underlying mental health conditions.

## Figures and Tables

**Table 1:** Samples used for applying the separation protocol.

Case	Sex	Age	Cause of Death	Postmortem Delay (h)
1	Male	46	Ischemia	5
2	Male	54	Myocardial infarct	5
3	Male	67	Ischemia	4
4	Male	69	Ischemia	16
5	Male	79	Ischemia	2
8	Male	81	Ischemia	10

**Table 2:** Designed primers for MSP.

Name	Sequence 5′-3′	Tm (°C)
GRM3-MS	TTT AGT ATT TTC GGA TGG GC	60.22
GRM3-MAS	CGC GAC TCA ACT ACA CAC TAC	60.14
GRM3-NMS	TAG TTT AGT ATT TTT GGA TGG GT	56.95
GRM3-NMAS	CTC ACA ACT CAA CTA CAC ACT ACA	58.5

**Table 3:** Control strategy for MSP.

Lane	Sample DNA	Primer	Sequence	Expected Amplicon (bp)	Observed Amplicon (bp)
1	Methylated commercial DNA	*In-house* primer designed to detect methylated sites.	MS (TTT AGT ATT TTC GGA TGG GC) MAS (CGC GAC TCA ACT ACA CAC TAC)	174	174
2	Methylated comercial DNA	Comercial primer designed to detect methylated sites.	Unknown	182	182
3	Non-Methylated comercial DNA	In-house primer designed to detect non-methylated sites.	NMS (TAG TTT AGT ATT TTT GGA TGG GT) NMAS (CTC ACA ACT CAA CTA CAC ACT ACA)	174	174
4	Non-Methylated comercial DNA	Comercial primer designed to detect non-methylated sites.	Unknown	254	254
5	Modified DNA obtained from isolated cells	In-house primer designed to detect methylated sites.	MS (TTT AGT ATT TTC GGA TGG GC) MAS (CGC GAC TCA ACT ACA CAC TAC)	174	174
6	No-DNA Sample (Water)	Comercial primer designed to detect methylated sites.	Unknown	0	0

## Data Availability

The datasets generated during and/or analyzed during the current study are not publicly available due to ethical and privacy restrictions.

## Supplementary Materials

The following supporting information can be downloaded at: https://www.mdpi.com/article/10.3390/mps8030061/s1, Detailed protocol and Figure S1: Methylation-Specific PCR.
