# Supplementary material for: A Refined Approach to Isolate Interneurons for High-Validity Epigenetic Studies in Human Brain Tissue
Source: Methods Protoc. 2025 Jun 5;8(3):61. doi: 10.3390/mps8030061 (PMC12196513; doi:10.3390/mps8030061)
Supplement: Supplementary file 1 [file mps-08-00061-s001.zip › mps-3624811-supplementary.pdf]

SUPPLEMENTARY MATERIAL

1. Detailed protocol. Keep in mind that Percoll® density could vary from different batches but it is relatively constant (around 1.125 – 1.135). Final density may be stablished according to manufacturer's instructions, but we consider that keeping percentages (% vol/vol) is easier to understand and to apply. Thus, the first step is to confirm that batch density is the same. In another case, the desired density can be easily recalculated. The density of the received Percoll ® batch used in this work was 1.129 g/ml (Sigma, Cat. P1644. Batch #SLCB3622). To avoid changes in PBS composition we acquired commercially (ThermoFisher, Cat. 14200-075)

A) Cell separation and fractioning.

1. Weight the chosen tissue by using a pre-weighted 1.5ml microtube. Use at least 0.1g of tissue. *Technical tip. Samples as small as 3 mg were also used but the final recovery of cells, after sorting, is extremely low.*

2. Cut the samples in thin slices by using a sterile blade.

3. Add 1 ml of Accutase™ solution (Sigma. Cat.A6964) and verify that this volume is enough to cover the slices. *Technical tip. You can use the blade to further chop the samples or triturate it by using sterile laboratory tweezers. When larger quantities of tissue should be processed it is a good procedure to divide them in fractions and apply at least 500ul of Accutase™ solution to each sample.*

4. Incubate the samples for 15 minutes at 4ºC. *Technical tip. It could be incubated without shanking at the lab fridge or by using a rotary shaker at 4ºC. Shaking should be always gently to avoid the degradation of cells and DNA spilling from them, given that this situation will complicate the purification or staining procedures.*

5. After incubation, apply the homogenate tissue through 100 microns pore diameter cell strainer (Falcon. Cat. 352360).

6. Recover the flow-thought and applied it through 40 microns pore diameter cell strainer (Falcon. Cat. 352340). *Technical tip: Use enough PBS 1X (Sigma. Cat. #D1408 10X) to facilitate the cell recovery, trying to avoid the retention of filters membranes.*

7. Centrifuge the flow-through obtained in the latter step for 2 minutes at 300x*g* in a swinging-bucket rotor centrifuge (Eppendorf Centrifuges. Cat. 5702). Separate the pellet and label it as Pellet A. Keep it on ice.

8. Centrifuge the supernatant for 2 minutes at 300x*g* in a swinging-bucket rotor centrifuge. Separate the pellet and label it as Pellet B. Keep it on ice.

9. Centrifuge the supernatant for 2 minutes at 1000x*g* in a swinging-bucket rotor centrifuge. Separate the pellet to a new polypropylene tube and label it as Pellet C. Keep it on ice cold. *Technical tip. The three aforementioned steps are focused on recovering the maximum of cells that may be retained by debris.*

10. Pool the three pellets as one ensuring a maximum volume of 3 ml and applied it to a 3ml 20% Percoll® column. Centrifuge for 3 minutes at 1000x*g* in a swinging-bucket rotor centrifuge. Separate the pellet and the supernatant fractions.*Technical tip. If volumes are larger, divide them and complete to 3ml by using PBS1X and applied the to an appropriate number of additional Percoll® columns.*

11. Label the tube containing the pellet as Pellet 1. Keep it on ice.

12. Directly centrifuge the obtained supernatant for 2 minutes at 1000x*g* in a swinging-bucket rotor centrifuge. Separate the pellet and the supernatant fractions.

13. Label the tube containing the pellet as Pellet 2. Keep it on ice. *Technical tip. This is pre-purification step, and to avoid the loss of sample we repeat the centrifuge step.*

13. Pool the two pellets as one and applied 300ul of these pellets onto 800ul 35% Percoll® column. Centrifuge for 30 minutes at 400x*g* in a fixed-angle rotor centrifuge (Eppendorf Centrifuges. Cat. 5427R).

14. Separate the pellet and label it as Pellet a. Keep it on ice.

15. Directly centrifuge all the obtained supernatant for 5 minutes at 1000x*g* in a fixed-angle rotor centrifuge. Separate the pellet and label it as Pellet b. Keep it on ice.

16. Directly centrifuge all the obtained supernatant for 5 minutes at 1000x*g* in a fixed-angle rotor centrifuge. Separate the pellet and label it as Pellet b. Keep it on ice. *Technical tip: The latter is an optional step intended to assure the maximum recovery of cells and nuclei.*

17. Pool the pellets as one, complete to 0.5-1ml with cold ice PBS 1X and proceed to flow cytometry staining. Also, staining with 0.4% Trypan Blue will help to visualize the isolated cells/nuclei and to have a quick view of the obtained yield. *Technical tip. Counting cells by using a Neubauer chamber it is useful for fine-tuning of flow cytometry techniques.*
